# Editorial: Pulmonary fibrosis and lung carcinogenesis: do myofibroblasts and cancer-associated fibroblasts share a common identity?

**DOI:** 10.3389/fonc.2024.1389532

**Published:** 2024-03-11

**Authors:** Daniela de Totero, Emanuela Barisione, Enrico Clini

**Affiliations:** ^1^ Molecular Pathology Unit, IRCCS Ospedale Policlinico San Martino, Genoa, Italy; ^2^ Interventional Pulmonary Unit, IRCCS Ospedale Policlinico San Martino, Genoa, Italy; ^3^ Respiratory Disease Unit, Department of Medical and Surgical Sciences, University of Modena and Reggio Emilia, University Hospital of Modena, Modena, Italy

**Keywords:** pulmonary fibrosis, lung cancer, extracellular matrix, myofibroblasts, cancer-associated fibroblasts

Pulmonary fibrosis (PF) can be considered as a peculiar form of Interstitial Lung Disease (ILD), including a high and heterogeneous number of subtypes, all sharing scarring of the lung tissue (1). Among ILDs, a large group of diseases of known and unknown aetiology is included, largely heterogeneous in term of course, clinical and radiological presentation, prognosis, and treatment (2).

The most common type of progressive lung fibrosis is the Idiopathic Pulmonary Fibrosis (IPF), where “idiopathic” indicates that the ultimate cause is unknown. Moreover, it has become evident recently that other several secondary ILD may develop progressive fibrosis of the lung, which more commonly occurs either in hypersensitivity pneumonitis, autoimmune diseases such as rheumatoid arthritis and systemic sclerosis, idiopathic nonspecific interstitial pneumonia, and rare forms of unclassifiable ILD (3, 4). Indeed, the diagnostic process for ILD can be very challenging, requiring a thorough medical history, physical examination, lung function tests, high resolution computed tomography (HRCT) of the lung, bronchoalveolar lavage and, if necessary, lung tissue examination (5, 6).

Interestingly, PF is a risk factor for developing lung cancer (LC) (7) and patients suffering from IPF have a five-fold increased risk compared with the general population (8). In turn, patients with lung cancer having or developing IPF typically have worse prognosis than those without (9). Multiple common genetic, molecular and cellular processes may connect lung fibrosis with lung cancer. Among these, the aberrant extracellular matrix (ECM) deposition by myofibroblasts, that progressively causes stiffness of the lung parenchima, and compromises local biochemical properties, appears to have a prominent role (10). The identification of specific markers and/or molecular pathways common to ILD-myofibroblasts as well as to cancer-associated fibroblasts could shed new lights on the mechanisms driving carcinogenesis. The recent advent of the so called “omic” approaches, such as genome-profiling or single-cells transcriptomic studies, represents a new way to explore biomarkers or signaling pathways involved in the development of lung cancer associated to PF, as well as to monitor temporal dynamic expression of specific gene along the disease.

With this Research Topic we aimed at collecting articles dealing with multiple aspects related to the potential shared pathogenesis between lung cancer and lung fibrosis. Overall, no studies to date clearly explain whether a strict relationship exists between the two conditions. Notwithstanding and based on a clinical ground, a growing interest in the role of comorbidities when studying IPF was recorded among researchers. Indeed, smoking habit, older age, male sex, and lung emphysema are all together risk factors for developing lung cancer (LC) in patients suffering from IPF, which therefore represents a potential driver for lung carcinogenesis in the individual patient (11). More recently, a canadian LC screening study has shown interestingly that around 4% of the included individuals had coexisting interstitial lung abnormalities at the CT scan, 2/3 of them localized close to the pleural wall (12).

Despite the origin of IPF is still unknown, similarly to LC, studying the cellular proliferative mechanisms, considered a key process leading to the lung tissue fibrotic transformation (10), might lead to discover new effective anti-fibrotic agents. In this light, there are currently two antifibrotic/antiproliferative drugs registered and used as a therapeutic strategy to treat patients with IPF, with the aim to slow-down the respiratory function decline and potentially to improve prognosis in the long-term (13, 14)

As recently emphasized, changes within the ECM proteome (matrisome), composed of the “core” ECM proteins (collagens, glycoproteins and proteoglycans of the ECM structure) and of ECM-associated proteins (mucins, enzymes, cytokines) affect lung tissue architecture, causing lung diseases and tumor development (15–18).

The quantitative proteomic approach performed by Titmarsh et al. to determine changes in matrisome ECM proteins between human non-small cell lung cancer (NSCLC) tissues and patient-matched not cancerous lung, suggests that the up-regulation of the collagen cross-linking enzyme peroxidasin (PXDN) and a disintegrin and metalloproteinase with thrombospondin motifs 16 (ADAMTS16) discriminates malignant from non-malignant lung tissue and appears associated to poorer survival. Trough proteomic profiling of formalin-fixed paraffin-embedded (FFPE) specimens of IPF lung tissues and controls, Samarelli et al. has highlighted that the over-expression of five proteins, such as lymphocyte cytosolic protein 1 (LCP1), peroxiredoxin-2 (PRDX2), transgelin 2 (TAGLN2), lumican (LUM) and mimecan (OGN) are associated with advanced disease stage and impaired pulmonary function, further suggesting that they may represent markers of fibrosis and carcinogenesis. Sahu et al., other than reviewing risk factors involved in lung squamous cell carcinoma (LSCC), pointed to the importance of developing and improving mouse models to identify novel biomarkers. A collagen-risk model predictive of lung adenocarcinoma prognosis, based on the signature expression pattern of five collagen genes has been deviced by Dong et al. The study by Fang et al. has addressed a genotipic shift between paired primary and post-operative recurrent tumors in patients with non small cell lung cancer (NSLC): Next Generation Sequencing analysis demonstrates a relative frequence of genetic shifts that may affect the impact of subsequent treatment outcome. Taken together, these manuscripts in the series underlay the complex key biologic/pathogenetic role played by several mediators in the lung fibrotic process.

From a clinical point of view the study from Li et al. documents that, when connective tissue diseases (CTDs) involving the lung coexists with cancer, the efficacy of the first-line Epidermal Growth Factor-Tyrosine kinase inhibitor therapy for lung adenocarcinoma is impaired, therefore highlighting the worse outcomes for patients with CTDs-LC: individualized therapy for these patients could be envisaged. Finally, the systematic review and meta-analysis performed by Wang et al. provides new evidence for a higher risk of acute exacerbation of ILDs in lung cancer patients under chemotherapy.

To conclude, the present Research Topic has extended our knowledge on the mechanisms related to the risk of developing lung cancer in patient suffering from ILDs or vice versa, also highlighting how these patients require special care(s) in managing an adequate therapeutic approach.

## Author contributions

DT: Conceptualization, Writing – review & editing, Data curation, Writing – original draft. EB: Conceptualization, Writing – review & editing. EC: Conceptualization, Supervision, Writing – review & editing.
